# Acknowledgement to Reviewers of *Biomedicines* in 2018

**DOI:** 10.3390/biomedicines7010009

**Published:** 2019-01-24

**Authors:** Biomedicines Editorial Office

**Affiliations:** MDPI, St. Alban-Anlage 66, 4052 Basel, Switzerland

Rigorous peer-review is the corner-stone of high-quality academic publishing. The editorial team greatly appreciates the reviewers who contributed their knowledge and expertise to the journal’s editorial process over the past 12 months. In 2018, a total of 114 papers were published in the journal, with a median time to first decision of 15.5 days and a median time to publication of 36.5 days. The editors would like to express their sincere gratitude to the following reviewers for their cooperation and dedication in 2018:
Ada, PopoloLu, JunAdamson, David CoryLundstrom, KennethAgrahari, VivekLupták, AndrejAgresti, AlessandraLutfy, KabirullahAhi, YadvinderMacabeo, Allan P.Airenne, KariMacNeill, AmyAl-Fatimi, MohamedMagni, FulvioAnas, Andrea RoxanneMaiolo, DanieleAngelici, GaetanoMalemud, Charles J.Angelino, DonatoMarceau, FrançoisAntipina, MariaMarnetto, FabianaAoki, KazunoriMartínez Iglesias, OlaiaAraniciu, CătălinMary, DidierBadr, ChristianMason, Jody M.Bamdad, CynthiaMcArdle, StephanieBarrero, Maria J.McClorey, GrahamBasak, DebasishMendonca, AntonioBawadekar, MandarMigita, ToshiroBeharry, Kay D.Mishra, Pawan KumarBelvisi, LauraMizuo, KeisukeBentel, Jacqueline M.Mohamedali, KhalidBentires-Alj, MohamedMorandi, LucaBertacchini, JessikaMoschetta, MicheleBodenstine, ThomasNatarajan, Sathish KumarBorgia, FrancescoNg, DominicBroadley, SimonNjoku, Dolores B.Brown, Anthony M.C.Ochieng, JosiahBrustmann, HermannOdland, Jon ØyvindBurger, MichaelOeckinghaus, AndreaCapasso, CristianOh, Daniel S.Carter, WayneOltra, ElisaCestmir, AltanerParashar, DeepakChalla, DilipPastor, FernandoChang, Kee-LungPaulmurugan, RamasamyChen, Nanhai G.Pedeux, RémyChina, ArnabPeigneur, SteveChoi, MinsigPelagalli, AlessandraChoudhary, SanjeevPelka, PeterClark, RichardPerkins, Neil D.Cortez, Maria AngelicaPerumal, SugunaCorthay, AlexandrePhesse, Toby J.Courtois, GillesPincus, SethCrabtree, JudyPinheiro, MarinaDabrowiak, JamesPochini, LorenaDaftarian, PirouzPreti, MarioDanesh-Sani, Seyed AmirPuglisi, FabioDangol, ManitaPunganuru, Surendra ReddyD’Argenio, ValeriaQasaimeh, Mohammad A.Deng, QingRamsay, Michael A.Deplazes, EvelyneRanjan, AlokDini, LucianaRennert, ChristianeDoekes, GertRicci, GiuliaElks, PhilRimondini, LiaEngelhart, AaronRinnerthaler, GabrielEssani, KarimRocha, SoniaExbrayat, Jean-MarieRochani, AnkitEzura, YoichiRodrigues, Celia F.Falanga, AnnaritaRoy, Dominic GuyFast, Loren D.Rundle, PaulFeng, YiRusso, GiuliaFitzgerald, DavidRuzza, PaoloFiume, GiuseppeRzymowska, JolantaFlavell, David J.Saha, ShyamaliForster, JamesonSaid, NeveenGarcía-Jiménez, CustodiaSarbini, ShahrulGarofalo, MariangelaSato-Bigbee, CarmenGautam, PrsonSatyam, AbhigyanGe, XiaodongSchödel, JohannesGea, AlfredoSebastian, AimyGentilucci, LucaSethi, GautamGilabert-Oriol, RogerShakibaei, MedhiGniewosz, MałgorzataShi, HuaGoel, PeeyushShungu, DikomaGómez De Cedrón, MartaSill, HeinzGonzález-Burgos, Elena MariaSingh, AnuragGorrell, MarkSingha Roy, SusmitGoyenvalle, AurélieSlominski, AndrzejGregoire, LaurentSmahel, MichalGudey, Shyam KumarSmith, Tim A. D.Gupta, VijayalaxmiSokoloski, KevinGurrapu, SreeharshaSolano, FranciscoH Shirvani, ArashSoliz, JorgeH Van Der Horst, EdwardSong, ChunhuaHailfinger, StephanSpingler, BernhardHamann, MartineSrivastava, SaurabhHammer, SuntreaStanley, Jone A.Hartman, MatthewStrack, PeterHatachi, YukimasaStrano, SabrinaHe, HongSu, Chia-HaoHellweg, ChristineSun, QianHernández-Alcoceba, RubénSun, WeiHildreth, BlakeSzakmany, T.Hoemann, Caroline D.Szumny, AntoniHollenstein, MarcelTabernero, María DoloresHolst, Peter JohannesTada, SeiichiHopkins, SamanthaTalk, Andrew C.Hu, Wen-YangTalukder, PoulamiIlmer, MatthiasTanaka, ToshiakiImbert, VéroniqueTazawa, HiroshiInoue, AtsukoTevosian, Sergei G.Ippoliti, RodolfoToietta, GabrieleIsola, GaetanoToiu, AncaIto, HiroyasuTorta, DianaJankowska, MilenaTosi, SolveigJeremy, Teoh Yuen ChunTrincone, AntonioJiang, ChengTseng, Chih-HuaJin, JiefuTsuchida, SachioJoseph, SusanUmar, Akhilesh KKajiya, HiroshiUrbanelli, LorenaKandimalla, RajuVaidyanathan, BharatKanzaki, HiroyukiValentovic, MonicaKapitsinou, PinelopiVamanu, EmanuelKarlic, HeidrunVan’t Wout, MaschaKarpiński, Tomasz M.Vassilopoulos, GeorgeKenny, Hilary A.Vellante, FedericaKhisamutdinov, EmilVelliyagounder, KabilanKietzmann, ThomasVicari, Enzo DanteKoga, FumitakaVostinaru, OliviuKoller, GabrieleWada, JunKonstantinov, Spiro M.Warner, SusanneKorfei, MartinaWatanabe, KazuhideKracht, MichaelWawrusiewicz-Kurylonek, NataliaKu, ZhiqiangWildemann, BrittKuçi, SelimWischhusen, JoergKuehl, MichaelWodrich, HaraldKumar, GauravWoo, Se JoonLang, DiXiang, DongxiLaPres, John J.Xiong, ZhaohuiLatchman, YvetteYagüe, ErnestoLauritano, DorinaYamamotova, AnnaLauschke, VolkerYoo, So YoungLebleu, BernardYu, Cheng-PingLee, KeehoonZabetakis, IoannisLee, Chia-HwaZakhari, SamirLee, Che-HsinZhang, Jian-minLee, Joo HyoungZhang, GuanshiLeporatti, StefanoZhao, RuimingLi, YeZhao, WenxiuLi, DeminZhou, HaiyanLin, Kwang-HueiZhu, YuwenLiu, MengranZorc, BrankaLöffler, Markus



